# CXCR4-CCR7 Heterodimerization Is a Driver of Breast Cancer Progression

**DOI:** 10.3390/life11101049

**Published:** 2021-10-07

**Authors:** Valentina Poltavets, Jessica W. Faulkner, Deepak Dhatrak, Robert J. Whitfield, Shaun R. McColl, Marina Kochetkova

**Affiliations:** 1 Centre for Cancer Biology, SA Pathology and the University of South Australia, Adelaide, SA 5000, Australia; valentina.poltavets@unisa.edu.au (V.P.); jessicafaulkner00@gmail.com (J.W.F.); 2 Surgical Pathology Unit, SA Pathology, Adelaide, SA 5000, Australia; deepak.dhatrak@sa.gov.au; 3 School of Medicine, University of Adelaide, Adelaide, SA 5000, Australia; Robert.Whitfield@sa.gov.au; 4 Breast, Endocrine and Surgical Oncology Unit, Royal Adelaide Hospital, Adelaide, SA 5000, Australia; 5 Department of Molecular and Cellular Biology, School of Biological Sciences, University of Adelaide, Adelaide, SA 5005, Australia; 6 Centre for Molecular Pathology, University of Adelaide, Adelaide, SA 5000, Australia

**Keywords:** chemokine receptors, heterodimer, CXCR4, CCR7, breast cancer

## Abstract

Metastatic breast cancer has one of the highest mortality rates among women in western society. Chemokine receptors CXCR4 and CCR7 have been shown to be linked to the metastatic spread of breast cancer, however, their precise function and underlying molecular pathways leading to the acquisition of the pro-metastatic properties remain poorly understood. We demonstrate here that the CXCR4 and CCR7 receptor ligands, CXCL12 and CCL19, cooperatively bind and selectively elicit synergistic signalling responses in invasive breast cancer cell lines as well as primary mammary human tumour cells. Furthermore, for the first time, we have documented the presence of CXCR4-CCR7 heterodimers in advanced primary mammary mouse and human tumours where number of CXCR4-CCR7 complexes directly correlate with the severity of the disease. The functional significance of the CXCR4-CCR7 association was also demonstrated when their forced heterodimerization led to the acquisition of invasive phenotype in non-metastatic breast cancer cells. Taken together, our data establish the CXCR4-CCR7 receptor complex as a new functional unit, which is responsible for the acquisition of breast cancer cell metastatic phenotype and which may serve as a novel biomarker for invasive mammary tumours.

## 1. Introduction

Breast cancer (BC) is the most common neoplasm in women worldwide, and metastatic mammary tumours account for over 40% of all cancer-related deaths in females. Progression of breast cancers from benign to invasive as well as metastatic forms is the main cause of cancer-related mortality in women and metastatic BC remains incurable [1]. Novel insights into the mechanisms of the invasive spread of BC are therefore imperative.

Chemokine receptors belong to the class A family of G-protein coupled receptors (GPCRs) and together with their respective ligands (chemokines), form a complex network that mediates multiple cellular functions in development, homeostasis, and pathology [2]. A significant line of evidence has been accumulated regarding the roles of chemokine receptors in cancer and metastasis. Cancer cells manipulate the chemokine system, either through upregulation of specific receptors or through secretion of chemokines to regulate cell migration, proliferation and survival in autocrine or paracrine fashions [3]. 

In cancer progression and metastasis, the expression of chemokine receptor 4 (CXCR4) and chemokine receptor 7 (CCR7) is of particular importance [4]. Since the first demonstration of their tumour-promoting role in breast cancer [5], the significant contribution of these receptors to the metastatic progression in a number of cancers is now firmly established [6,7,8,9,10]. Furthermore, the expression of both CXCR4 and/or CCR7 are of significant prognostic value in multiple other cancers. Most importantly, both CXCR4 and CCR7 have been proposed to be potential therapeutic targets in cancer treatments [11].

GPCR aggregation has been outlined as a critical determinant of their signalling and function. These seven transmembrane receptors have been shown to form homodimers, heterodimers, and multimeric complexes [12]. Importantly since the first demonstration of the pathologic association between the angiotensin II AT1 receptor and the bradykinin B2 receptor [13], GPCR aggregates were shown to strongly contribute to many disease conditions such as atherosclerosis, Alzheimer’s disease, neurodegeneration and others [14]. 

Regulation of chemokine receptor activity was also found to be at least partially dependent on the formation of homo- and heterodimers [15]. Chemokine receptor dimerization affects ligand affinity, downstream signalling and receptor internalisation/recycling [16]. Notably, accumulating evidence indicates that the receptor heterodimer is an active unit with distinct and unique signalling as well as pharmacological properties [17,18,19,20] and that dimeric or oligomeric chemokine receptor complexes should be considered as unique signal-transducing units with their own distinct biochemical and functional signatures [21]. 

The association of chemokine receptors in heterocomplexes has been well documented. Heterodimerization and the functional outcomes of CXCR4 and CCR7 cross-regulation have been mainly reported in the context of immune cells. In CD4^+^ T cells, CXCL12-mediated signalling promotes CXCR4-CCR7 heterodimerization and further augments T cell migration [22]. During B cell development, CXCR4 and CCR7 association results in deficient activation of the G protein alpha subunits Giα1 and 2 [23], leading to a differential downstream signalling specific to the heterodimer. The co-stimulatory effect of CXCR4 and CCR5 in primary T cells has been correlated to the formation of CCR5-CXCR4 heterodimers, with distinctive signalling and biological properties [18]. Furthermore, CXCR4 and ACKR3 co-expression result in constitutive recruitment of β-arrestin-2. It also enhances cell migration, in response to CXCL12 stimulation and lung metastasis of breast cancer cells [24,25]. 

However, despite these previous findings, due to the inherent difficulty in detecting native chemokine receptor dimers, as well as the limitations in the availability of in vivo models, there is minimal evidence in relation to the functional outcomes of chemokine receptor heterodimerization in a pathological context in general, and in cancer in particular [16,26]. 

We have previously demonstrated that it is the functional activation of both CXCR4 and CCR7, as opposed to their expression levels, that correlates with the invasive and metastatic phenotype of breast cancer cells [27]. We also established a direct connection between the activation of CXCR4 and CCR7, and the inhibition of detachment-induced apoptosis (anoikis) in metastatic breast cancer cells that potentially contributes to the metastatic spread of mammary tumours [28]. However, a possible physical association between CXCR4 and CCR7 or its functional significance in relation to breast cancer progression has not been previously investigated. In this study, the existence of the CXCR4-CCR7 heterodimers in primary mouse and human mammary tumours is shown for the first time. Moreover, we also demonstrate the significance of the CXCR4-CCR7 complex formation to tumour-promoting receptor function in breast cancer cells. The results described here may thus present new therapeutic opportunities by disrupting the CXCR4-CCR7 hetero-complex in the treatment of advanced breast cancer. 

## 2. Materials and Methods

### 2.1. Mice

All experimental procedures were approved by the animal ethics committee of the University of Adelaide. Mice were maintained in pathogen-free conditions in the University of Adelaide Animal Services facility. The FVB/NJ MMTV-PyMT mice were purchased from the Jackson Laboratory and were backcrossed for 14 generations to the mice with C57Bl/6 background. The C57Bl/6 background was subsequently confirmed by microsatellite analysis. 

### 2.2. Human Tissues

Ethical approval was granted by the Royal Adelaide Hospital Ethics Committee. Normal breast and carcinoma tissues were obtained from R. Whitfield (Breast Endocrine and Surgical Oncology Unit, Royal Adelaide Hospital). All patients gave written informed consent for use of tissue for medical research prior to surgery. The human breast tissue microarray (TMA-1005) was purchased from Protein Biotechnologies (Ramona, CA, USA). 

### 2.3. Human Gene Expression Analysis

The gene expression dataset used here was METABRIC [29]. Raw data were obtained from the Oncomine™ platform (Thermo Fisher Scientific, Waltham, MA, USA) for *CXCR4* (GC02M136114) and *CCR7* (GC17M040556). A compound log_2_ fold-change gene profile for the two-gene expression was created by taking a mean log_2_ fold change of each individual gene [30]. Statistical analysis was performed using GraphPad Prism software (San Diego, CA, USA). 

### 2.4. Cell Lines

All human cell lines were purchased from the American Type Culture Collection (Manassas, VA, USA): MDA-MB-231 (CRM-HTB-26); MDA-MB-361 (HTB-27); T47D-KBluc (CRL-2865); MDA-MB-453 (CRL-1500); ZR-75-30 (CRL-1504). Cells were grown at 37 °C in 5% CO_2_, in a humidified atmosphere according to the supplier’s instructions. 

### 2.5. Isolation of Mouse Mammary Epithelial Cells

Total mammary cells were derived as previously described [31]. Total cell populations were isolated from multiple lesions and pooled from 2–3 mice. Briefly, all mouse mammary glands were dissected, and lymph nodes removed, manually dissociated and then digested in Dulbecco’s modified Eagle’s medium (DMEM) (Thermo Fisher Scientific, Waltham, MA, USA) supplemented with 1 mg/mL collagenase IA, 100 U/mL hyaluronidase (Worthington Biochemical Corporation, Lakewood, NJ, USA) and 2% foetal calf serum (FCS) for three h at 37 °C. The freshly isolated total cell preparations were cultured overnight in non-adherent conditions [1:1 volumes of DMEM and Ham’s F12 nutrient mix (Thermo Fisher Scientific), supplemented with 10 ng mL^−1^ epidermal growth factor (EGF), 20 ng mL^−1^ basic fibroblast growth factor (bFGF) (both PeproTech, Cranbury, NJ, USA) and 0.5 × B27 (Thermo Fisher Scientific)] to obtain a pure epithelial cell culture. Cells were then cultured adherently in DMEM supplemented with 10% FCS and antibiotic–antimycotic (Thermo Fisher Scientific) at 37 °C in 5% CO_2_ in a humidified atmosphere. 

### 2.6. Isolation of Human Mammary Epithelial Cells

Isolation of the human mammary epithelial cells from surgical specimens were performed as previously reported [32]. Briefly, human specimens were manually dissociated and digested in DMEM supplemented with 20 mM HEPES (Thermo Fisher Scientific, Waltham, MA, USA), 1 mg/mL collagenase IA, 100U/mL hyaluronidase (both Worthington Biochemical Corporation, Lakewood, NJ, USA), 12 U/mL DNase I (Merck, Darmstadt, Germany), 1% penicillin-streptomycin and 0.25 μg/mL fungizone (both Sigma Aldrich, St. Louis, MO, USA). Initial digests were washed with DMEM, lysed of red blood cells and single-cell suspensions were obtained by further 10 min digest in trypsin (Thermo Fisher Scientific) at room temperature. Cells were filtered through a 70 μm nylon mesh (Corning, Somerville, MA, USA) prior to further analysis or culture.

### 2.7. In Vivo Metastasis Assay

An experimental metastasis experiment was carried out as previously described in [28]. Briefly, human breast cancer cell lines were engineered by retroviral transduction to stably express GFP. Six to eight-week female CB-17 SCID mice (ARC, Perth, WA, Australia) were injected IV into the tail vein with 5 × 10^5^ cells suspended in 200 µL PBS (Thermo Fisher Scientific, Waltham, MA, USA). Ten weeks after cell injection mice were sacrificed, lungs excised, perfused with PBS and bright field and fluorescent images were recorded by stereo microscope Leica MZ16FA (Wetzlar, Germany).

### 2.8. Ligand Cooperation Assay

Synthetic chemokine ligands were obtained from the Biomedical Research Centre, University of British Columbia (Vancouver, BC, Canada). Cells in suspension were incubated with 5 ng/mL of biotinylated CCL19 alone or in combination with 10 ng/mL of unlabelled CXCL12 for 30 min on ice. Cells were fixed in 4% formaldehyde (Sigma Aldrich, St. Louis, MO, USA), incubated with FITC-conjugated streptavidin (Rockland Immunochemicals, Limerick, PA, USA) and analysed by flow cytometry. 

### 2.9. Flow Cytometry

5 × 10^4^ cells were fixed in 4% formaldehyde (Sigma Aldrich, St. Louis, MO, USA) and immunostained for 45 min on ice in PBS containing 0.5% bovine serum albumin (PBS-0.5% BSA). The antibodies used, were PE-conjugated anti-human CXCR4 (clone 1D9, BD, Franklin Lakes, NJ, USA), biotinylated anti-mouse CXCR4 (clone 2B11, BD, Franklin Lakes, NJ, USA) and APC-conjugated anti-human/mouse CCR7 (clone 3D12, eBioscience, Thermo Fisher Scientific, Waltham, MA, USA). Samples containing biotinylated antibodies were treated with PE-conjugated streptavidin (Rockland Immunochemicals, Limerick, PA, USA) in PBS/0.5% BSA for 30 min on ice. Flow cytometry was carried out using FACSCanto equipment (BD, Franklin Lakes, NJ, USA) with standard settings. Data analysis was performed using FlowJo software (BD, Franklin Lakes, NJ, USA). Positive events were defined above the level of background staining observed using matched isotype control antibodies. 

For the FACS-FRET experiments, the bandpass filter settings for detection on FACSCanto equipment were changed to the following excitation/emission windows: PE—488 nm/>556LP + 585 ± 42; FRET—488 nm/>655 nm, and APC—633 nm/660 ± 20. Positive FRET signal was defined as the level of fluorescence above background staining observed using matched isotype control antibodies. 

### 2.10. Immunofluorescence Analysis 

For the human tissue analysis, formalin-fixed, paraffin-embedded sections of 4 µm were rehydrated and immersed in a 10 mM citric acid buffer at pH 6.0, boiled for 20 min, then cooled to room temperature. Specimens were blocked in 5% normal goat serum in PBS for 30 min and incubated overnight at 4 °C with the mouse anti-human CXCR4 (clone 44708, R&D Systems, Minneapolis, MN, USA) at 20 μg/mL; rabbit anti-human CCR7 (clone Y59, Epitomics, Burlingame, CA, USA) at 15 μg/mL diluted in 0.5%BSA in PBS. The secondary antibodies used, were anti-mouse Alexa Fluor® 647 and anti-rabbit Alexa Fluor® 488 (Invitrogen, Thermo Fisher Scientific, Waltham, MA, USA) at 1:400 dilution and were then incubated for 90 min at room temperature in the dark. Samples were mounted using Vectashield mounting media (Vector Laboratories, Burlingame, CA, USA). Immunofluorescence images were acquired using the Leica SP5 spectral scanning confocal microscope (Wetzlar, Germany). 

### 2.11. Proximity Ligation Assay (PLA) 

Following the primary antibody incubation described in immunofluorescence analysis, the PLA procedure was carried out according to the manufacturer’s protocol (OLINK Bioscience, Uppsala, Sweden). Briefly, primary antibodies of two different species are bound to the target proteins. Next, the oligonucleotide-conjugated secondary antibody pairs (PLA probes) are added. The close proximity of bound PLA probes brings together two oligonucleotides from both probes as a template for complementary circular DNA ligation. Finally, DNA polymerase addition initiates rolling circle amplification (RCA) primed by oligonucleotides on one of the PLA probes, and fluorescent oligonucleotides are used to visualize the RCA products. RCA product consists of a single DNA strand with several hundred complements of the DNA circle that is labelled by hybridized fluorophore-conjugated short DNA oligonucleotides (detection oligonucleotides). The bright discrete RCA product consists of a distinct sub-μm signal that allows visualization and enumeration of single molecules by fluorescent microscopy [33,34].

Detection reagent Red was used for the amplification step. After the final PLA step, the slides were washed with PBS and further incubated with anti-human EpCAM Alexa Fluor® 488 (Santa Cruz Biotechnology, Dallas, TX, USA) at 1:50 dilution in 0.5% BSA in PBS overnight. Subsequently, samples were washed and mounted with Vectashield mounting media (Vector Laboratories, Burlingame, CA, USA) for TMA samples and Vectashield mounting media containing DAPI for primary normal breast and tumour tissues. Images were acquired using the Leica TCS SP5 confocal microscope (Wetzlar, Germany). The slides were evaluated by sequential scanning. Ten fields of view were acquired for each TMA tissue spot. The number of PLA signals per μm^2^ of epithelium defined by the EpCAM positive staining were quantified using particle analysis in FIJI, (http://fiji.sc/Fiji, accessed on 15 August 2021). The images were processed by blind analysis. Representative images shown are the maximum intensity Z-projections.

For the human cell lines, one day prior to primary antibody staining, cells were plated onto Poly-L-Lysine adhesion slides (Thermo Fisher Scientific, Waltham, MA, USA) using 12-well tissue culture inserts (flexiPERM®, SARSTEDT, Nümbrecht, Germany) at 1 × 10^3^ cells per well. The cells were fixed using a 4% formaldehyde solution (Sigma Aldrich, St. Louis, MO, USA). The PLA procedure was then carried out as described above. Five fields of view for each cell line type were acquired. The number of PLA signals per cell were quantified using particle analysis in FIJI (http://fiji.sc/Fiji, accessed on 15 August 2021). Representative images shown are the maximum intensity Z-projections.

### 2.12. Forced Heterodimerization

Inducible CXCR4 and CCR7 dimerization system was constructed using iDimerize™ Inducible Heterodimer System (Clontech, Takara Bio Inc, Kusatsu, Shiga, Japan) according to manufacturer’s instructions. The system uses DmrA and DmrC domains altered to specifically bind the A/C heterodimerizer (ACH) ligand. Briefly, to generate the fusion pair, CXCR4 cDNA and Myc-tagged CCR7 cDNA was PCR-amplified and inserted into pHet-Mem1 (pCXCR4-H) and pHet-1 (pCCR7-M) plasmids. Primer sequences and vector maps are available upon request. 

### 2.13. Transient Transfection of Human Cell Lines 

MDA-MB-231 and T47D cell lines were transiently transfected with pCXCR4-H and pCCR7-M plasmids using Optifect™ transfection reagent (Invitrogen, Thermo Fisher Scientific, Waltham, MA, USA) according to the manufacturer’s instructions. Following transfection, the cells were cultured at 37 °C for 48 h prior to further analyses.

### 2.14. AlphaScreen cAMP Assay 

cAMP levels were assessed using the AlphaScreen Detection Kit (Perkin Elmer, Waltham, MA, USA). All cells were serum-starved for at least three hrs prior to the analysis. Briefly, cells were resuspended at 5 × 10^5^ cells/ml in AlphaScreen stimulation buffer containing HBSS (Thermo Fisher Scientific, Waltham, MA, USA) supplemented with 0.5 mM IBMX, 5 mM HEPES, 0.1% BSA (all from Thermo Fisher Scientific, Waltham, MA, USA), pH 7.4 in the presence or absence of forskolin (10 μM final concentration) (Sigma Aldrich, St. Louis, MO, USA) and treated or untreated with A/C heterodimerizer (100 mM final concentration). 3000 cells/well were aliquoted in triplicate into 384-well OptiPlate (Perkin Elmer, Waltham, MA, USA) and stimulated with CXCL12 and CCL19 chemokine ligands at indicated concentrations for 15 min at 37 °C. Further, cells were processed according to the manufacturer’s protocol. Alpha Screen signal was recorded using PHERAstar® FSX detection system (BMG Labtech, Ortenberg, Germany) using the AlphaScreen optical module (Ex. 680 nm/Em. 520−620 nm). Data analysis was performed using GraphPrism software (San Diego, CA, USA). 

### 2.15. Matrigel Invasion Assay 

MDA-MB-231 and T47D cells were co-transfected with pCXCR4-H and pCCR7-M iDimerize plasmids. 48 hrs post-transfection cells were serum-starved for at least three h and then treated or untreated with A/C heterodimerizer (100mM final concentration) for 1 hour at 37 °C. 5 × 10^4^ cells were then aliquoted in duplicate into Matrigel (BD, Franklin Lakes, NJ, USA) pre-coated 96 transwell assay plates with 5μm pore permeable support inserts (Corning, Somerville, MA, USA). Cells were then treated with CXCL12 at 10 ng/mL and/or CCL19 at 20 ng/mL final concentrations and allowed to migrate for 24 h at 37 °C. Cells were then detached from the underside of the transwell insert with 0.1% trypsin (Thermo Fisher Scientific, Waltham, MA, USA), loaded with Calcein AM/1 (Invitrogen, Thermo Fisher Scientific, Waltham, MA, USA) and cell density was estimated by PHERAstar® FSX detection system (BMG Labtech, Ortenberg, Germany) relative to total cell input.

### 2.16. Statistical Analysis

The statistical analysis was performed using GraphPad Prism software (San Diego, CA, USA). Refer to figure legends for details of the statistical analyses undertaken. P-values were calculated to assess statistical significance with levels of significance * *p* ≤ 0.05, ** *p* ≤ 0.01, *** *p* ≤ 0.001 and **** *p* ≤ 0.0001.

## 3. Results

### 3.1. CXCL12 and CCL19 Synergize in the Cell Surface Binding and Signaling Response Only in Invasive Breast Cancer Cells

Our previous results have demonstrated a link between both CXCR4 and CCR7 functional activation and the metastatic potential of breast cancer cells [28]. Considering the significance previously assigned to the GPCR association for their specific activity, we hypothesised that CXCR4 and CCR7 may heterodimerize to exert their pro-invasive function in mammary tumours. To establish the metastatic potential of the panel of human breast cancer cell lines used in our study, we tested their ability to form experimental metastasis in SCID mice. MDA-MB-231 and MDA-MB-361 cells were designated as ‘invasive/metastatic’ whilst MDA-MB-453, ZR 75-30 and T47D were assigned as ‘non-invasive/non-metastatic based in this test (Supplementary Figure S1A). 

**Figure 1:**
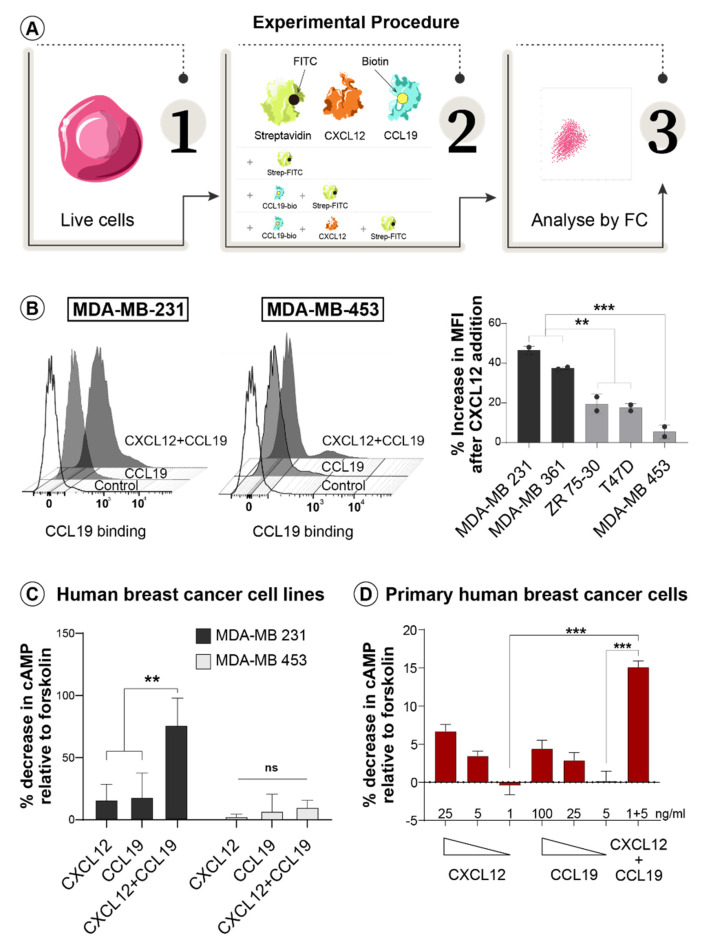
CXCL12 and CCL19 cooperate in cell surface binding and signalling responses selectively in invasive breast cancer cells. (**A**) Schematic representation of the cooperative ligand binding assay. (**B**) Cells were left untreated as (negative control) or were exposed to biotinylated CCL19 alone or in combination with CXCL12 as indicated followed by the addition of FITC-conjugated streptavidin and flow cytometry (FC) analysis. Shown are representative histograms for invasive (MDA-MB-231) and non-invasive (MDA-MB-453) cells (left panel). The increase in FITC MFI in cells treated with the combination of CCL19 and CXCL12 relative to cells treated with CCL19 alone was quantitated for a panel of cell lines and graphed (right panel). (**C**,**D**) cAMP relative concentration was assessed in lysates from breast cancer cell lines (**C**) or primary human breast tumour cells (**D**) that were pre-incubated with forskolin and then stimulated with CXCL12, CCL19 or their combination as indicated. All data shown are mean ± SEM with two-tailed student *t*-test and are representative of at least two independent experiments. Levels of significance ** *p* ≤ 0.01, *** *p* ≤ 0.001, ns—not significant.

As cooperative ligand binding has been reported in chemokine receptor dimers [35], we assayed the ability of CXCL12 to potentiate CCL19 interaction with its receptor in the panel of breast cancer cell lines with varied invasive properties. The binding of biotinylated CCL19 with or without CXCL12, to the live cells, were assayed by flow cytometry after the addition of FITC-conjugated streptavidin (Figure 1A). Results from this assay demonstrate that the addition of CXCL12 significantly increased the ability of CCL19 to bind to the cell surface, specifically in metastatic MDA-MB-231 and MDA-MB-361 cells (Figure 1B), suggesting a potential interaction between the two receptors. CXCR4 and CCR7 surface expression, measured by flow cytometry, was similar between the breast cancer cell lines, indicating that observed differences in ligand cooperativity in metastatic vs non-metastatic cells is not due to the differences in levels of receptor expression (Supplementary Figure S1B).

We next assessed the ability of CCL19 and CXCL12 to cooperate in eliciting a functional response. The inhibition of the cyclic AMP (cAMP) was chosen as a readout for this analysis as the predominant signalling pathway for Gi-coupled receptors, such as chemokine receptors, is adenylyl cyclase inhibition that leads to a reduction in intracellular cAMP levels [36]. Cells were pre-treated with forskolin to induce cAMP production (Supplementary Figure S1C) before the addition of CCL19, or CXCL12, or the two in combination. Subsequent assessment of cAMP levels in cell lysates demonstrated that singularly, both CXCL12 and CCL19 were able to weakly inhibit forskolin-induced cAMP production in invasive MDA-MB-231 cells, while their combination reduced cAMP concentration by more than 60% (Figure 1C). No changes to cAMP inhibition were detected in non-invasive MDA-MB-453 cells after the addition of either CXCL12, CCL19 alone or in combination (Figure 1C). Thus, these chemokine ligands not only bind cooperatively to the cell surface, but they also synergize in stimulating receptor function, specifically in metastatic breast cancer cells. 

To further establish the relevance of the above findings to human breast cancer, we examined the ability of CXCL12 and CCL19 to induce a cooperative functional response in primary cells from an invasive human mammary tumour. Mammary epithelial cells were purified from freshly excised patient tissue of grade three invasive ductal carcinoma and then expanded ex vivo. The ability of CXCL12, CCL19 or their combination to functionally activate their cognate receptors was then evaluated by measuring the inhibition of forskolin-induced cAMP production (Supplementary Figure S1D). We observed dose-dependent cAMP inhibition after the stimulation of cells with either ligand, demonstrating that CXCR4 and CCR7 are functionally active in advanced human breast cancer cells (Figure 1C). Most importantly, we found that CXCL12 and CCL19 also elicit a strong synergistic response in these cells when combined, even at concentrations suboptimal for individual responses, indicating that these chemokines cooperate functionally. This finding provided initial evidence for potential heterodimeric interaction between the CXCR4 and CCR7 receptors in invasive breast cancer, leading to further investigation.

### 3.2. CXCR4 and CCR7 Interact on the Surface of Invasive Breast Cancer Cells to Form Functional Heterodimeric Receptors

**Figure 2:**
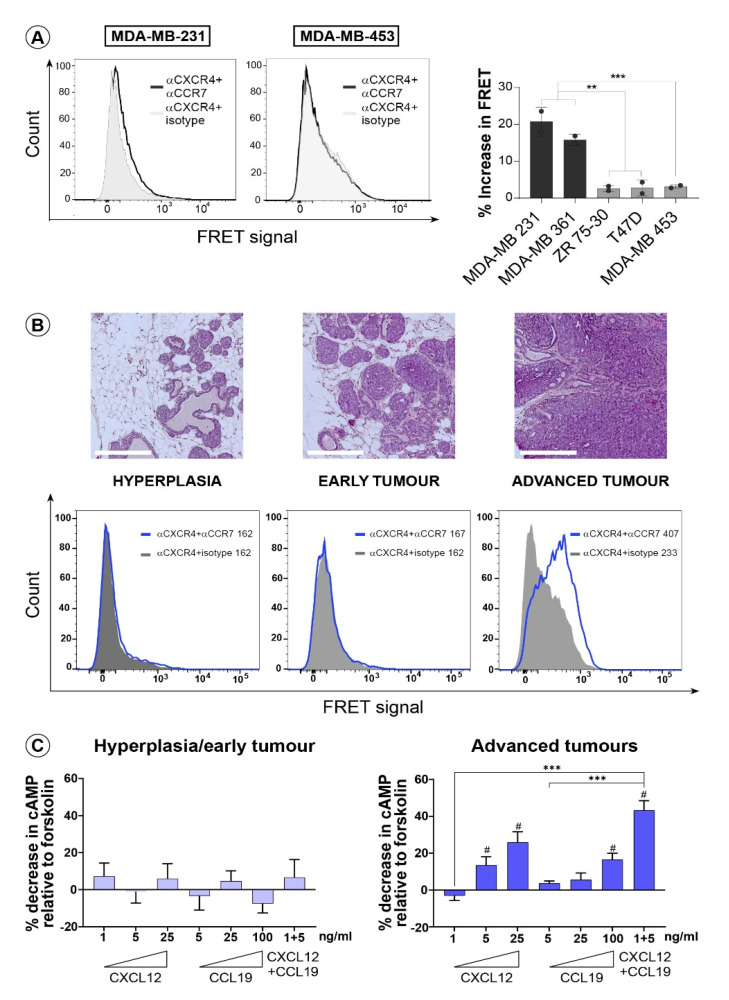
**Invasive status of breast cancer cells correlates with CXCR4 and CCR7 heterodimerization.** (**A**) FACS-FRET analysis was performed on a panel of human breast cancer cell lines. Shown are representative histograms for the invasive (MDA-MB-231) and non-invasive (MDA-MB-453) cells (left panel). Percent (%) increase in FRET MFI was calculated and graphed for each individual cell line. (**B**) H&E images of tissue sections (top, scale bar = 250 µm) and representative flow cytometry histograms with corresponding MFI values of FACS-FRET performed on epithelial tumour cells (bottom) from mouse mammary glands excised from PyMT transgenic animals at three distinct stages in cancer development. (**C**) Relative cAMP concentration was assessed in lysates from primary mammary mouse epithelial cells purified from early (left) or advanced (right) PyMT tumours that were pre-incubated with forskolin and then stimulated with CXCL12, CCL19 or their combination as indicated. All data shown are mean ± SEM, two-tailed student *t*-test and are representative of at least two independent experiments. Levels of significance ** *p* ≤ 0.01, *** *p* ≤ 0.001, #—significant decrease in cAMP relative to untreated cells.

To confirm the existence of the CXCR4-CCR7 complex we initially attempted to co-immunoprecipitate these receptors from the MDA-MB-231 cell lysates. Despite an extensive optimisation process, we failed to detect any CXCR4-CCR7 complexes using this approach. Since previous studies on GPCR oligomerisation have documented an important role of receptor allostery for their homo- and heterodimerization [37], we decided to employ in situ dimerization assay methods, that allow detection of receptor complexes on an intact cell membrane. The time-resolved fluorescence energy transfer (FRET) approach was selected as it permits the detection of interacting protomers within their native environment in intact cells and tissues. To assay CXCR4-CCR7 interaction in intact cells, we employed FRET coupled with flow cytometric analysis (FACS-FRET) (Supplementary Figure S2A) [38]. We used the FRET fluorophore pair of Phycoerythrin (PE) as a donor and Allophycocyanin (APC) as an acceptor, which were directly conjugated to anti-CXCR4 and anti-CCR7 monoclonal antibodies respectively, or APC-conjugated IgG as a control, to assess a potential native complex formation between these two receptors in our panel of breast cancer cells. The CXCR4 and CCR7 association was estimated as an increase in mean fluorescence intensity (MFI) in the FRET channel in cells incubated with antibodies for both receptors over that of cells incubated with the anti-CXCR4 and the control IgG. We detected a significant increase in the specific FRET signal over the control in invasive breast cancer cell lines only (Figure 2A), suggesting that the CXCR4 and CCR7 association may be linked to the invasive breast cancer phenotype. To provide further support to the notion that there exists a correlation between the invasive phenotype of breast cancer cells and the CXCR4-CCR7 complex formation, we investigated their association ex vivo in primary mammary tumour cells from the MMTV-PyMT mouse model that closely recapitulates stage-wise development of human breast cancer. To achieve this, we harvested epithelial tumour cells from three distinct stages of mammary cancer development and analysed the cells by FACS-FRET (Figure 2B, bottom panel). The correct “staging” of mammary lesions as hyperplasia ~8 weeks, early tumour ~11 weeks and advanced invasive tumour ~18 weeks was confirmed by histological examination of the H&E-stained tissue sections (Figure 2B, top panel). Strikingly, we found that a significant CXCR4 and CCR7 association was only detected in cells from the advanced primary tumours, whereas receptor interaction was practically absent in tumour cells from hyperplasia and early tumour stages. Importantly, expression levels of CXCR4 and CCR7 in all cells were very similar in all stages, as was determined by flow cytometry analysis on the same populations of primary mammary mouse cells that were used for the FACS-FRET assay (Supplementary Figure S2B). To begin testing for the functional significance of native CXCR4-CCR7 complexes in breast cancer, we assessed the ability of CXCL12, CCL19 or in combination to inhibit the forskolin-induced increase in cAMP production in MMTV-PyMT primary mammary cells (Supplementary Figure S2C). Epithelial cells from early tumours were unresponsive to any treatment suggesting that neither CXCR4 nor CCR7 in these cells can transduce the signal from their ligands (Figure 2C, left panel). However, treatment of cells from advanced tumours with either ligand led to significant inhibition of the forskolin-induced cAMP in a dose dependent manner (Figure 2C, right panel). Most importantly, treatment of the invasive mammary tumour cells with the combination of CXCL12 and CCL19 at suboptimal concentrations, elicited a very strong synergistic response in inhibiting cAMP, paralleling results obtained in cells from advanced primary human breast cancer. Thus, these results provide primary evidence for the existence of the CXCR4-CCR7 heterodimeric receptor in mammary tumours in vivo. Moreover, the data indicate that this complex may be a prerequisite for the activation of the tumour-promoting signalling downstream of these receptors in later stages of breast cancer progression.

### 3.3. CXCR4 and CCR7 Association Marks Breast Cancer Progression to Invasive Disease

**Figure 3:**
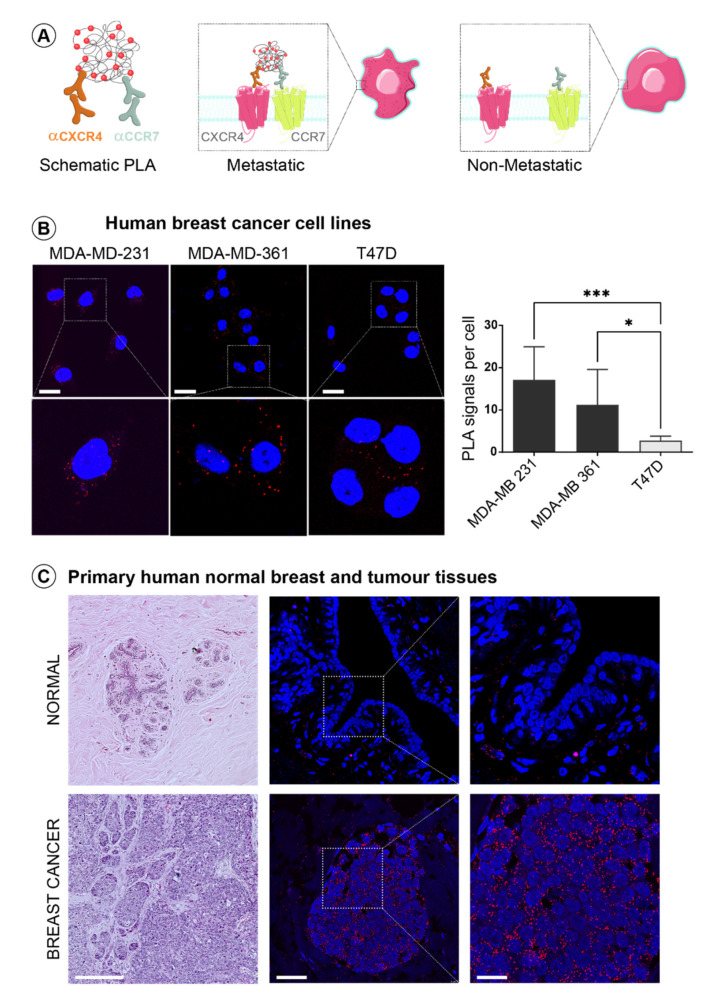
**CXCR4-CCR7 dimeric complexes are detected by proximity ligation assay.** (**A**) Schematic representation of the proximity ligation assay (PLA). Primary antibodies of two different species are bound to the target proteins; next, the oligonucleotide-conjugated secondary antibody pairs (PLA probes) are added to provide a template for circular DNA ligation. Finally, DNA polymerase addition initiates rolling circle amplification (RCA) and the final product consists of a distinct sub-μm signal that allows visualization and enumeration of single molecules by fluorescent microscopy. (**B**) CXCR4-CCR7 heterodimerisation was assessed by the PLA in invasive MDA-MB-231, MDA-MB-361 and non-metastatic T47D human breast cancer cell lines. Shown are representative images (scale bar = 25 µm) with zoomed-in areas, red - PLA signals, blue- cell nuclei, together with quantitative analyses of the number of PLA signals per cell. Data shown are mean ± SEM, two-tailed student *t*-test and are representative of at least three independent experiments. (**C**) Representative H&E images of normal breast and breast cancer tissue sections (left, scale bar = 250 µm) with corresponding immunofluorescence images of PLA (middle, scale bar = 50 µm). Image inserts are zoomed-in areas showing distinct PLA signals (red) and DAPI (blue) (right, scale bar = 20 µm). Levels of significance * *p* ≤ 0.05, *** *p* ≤ 0.001, ns - not significant.

**Figure 4:**
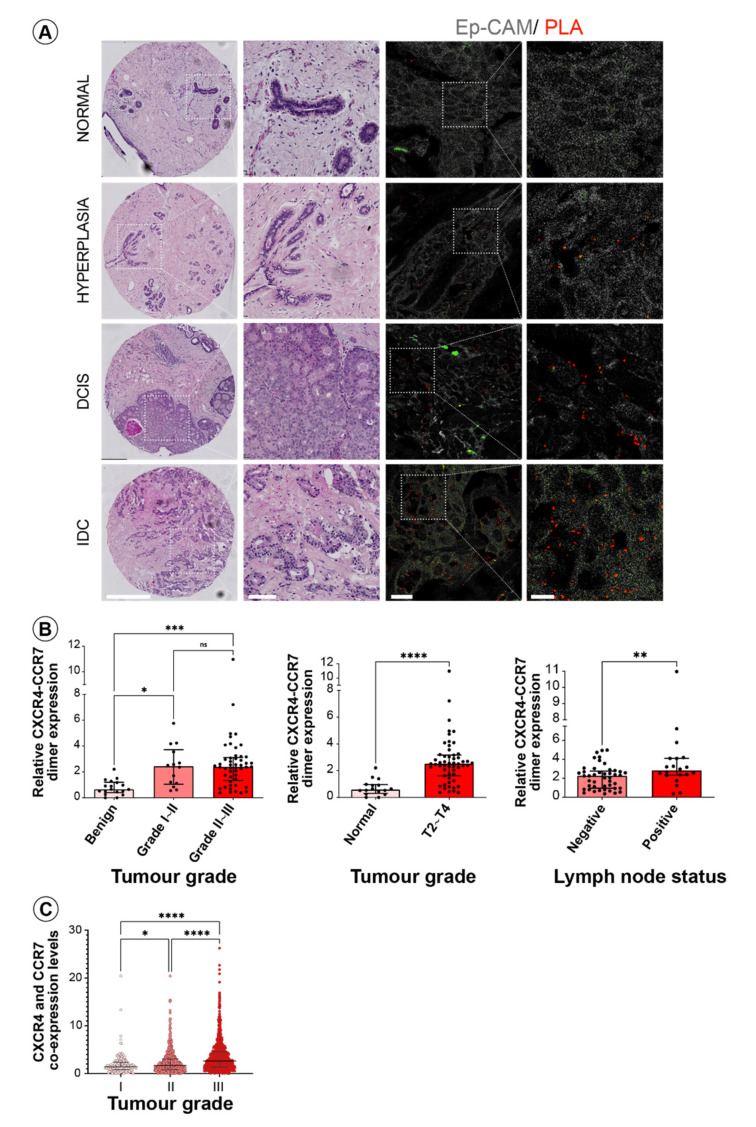
**CXCR4-CCR7 heterodimer is a biomarker for breast cancer progression.** (**A**–**C**) CXCR4-CCR7 dimer expression was assessed by PLA using a primary human breast tissue microarray. Shown are (**A**) representative H&E images with zoomed-in areas of representative tissue cores from normal, hyperplastic, ductal carcinoma in situ (DCIS) and invasive ductal carcinoma (IDC) (scale bar = 500 µm; zoomed-in areas scale bar = 100 µm) and corresponding immunofluorescence images of PLA (red—PLA signals, an epithelial area delineated by co-staining with anti-EpCAM antibody—grey). Scale bar = 180 µm; zoomed-in areas scale bar = 60 µm. (**B**) Number of PLA signals relative to the area of the epithelium was quantified and plotted against tumour grade (left), stage (middle), and lymph node status (right) of the tissues represented in the TMA. Data shown are median ± IQR, with unpaired Kruskal–Wallis (left) and Mann–Whitney (middle, right) tests. (**C**) CXCR4 and CCR7 gene co-expression was evaluated by assessing the median two-gene expression signature from the publicly available METABRIC dataset (N = 2136). Data shown are median ± IQR with unpaired Kruskal-Wallis test. Levels of significance * *p* ≤ 0.05, ** *p* ≤ 0.01, *** *p* ≤ 0.001 and **** *p* ≤ 0.0001, ns—not significant.

To determine if CXCR4 and CCR7 heterodimerization are relevant to the development of human breast cancer, we took advantage of the proximity ligation assay (PLA), a well-established method for determining in situ protein-protein interactions [39] (Figure 3A). The advantage of this method is that it allows the detection of minimally expressed native receptor heterodimers in endogenous settings. We first validated the PLA approach for the detection of endogenous CXCR4 and CCR7 association in breast cancer cells lines, differing in their invasive phenotype (Figure 3B). We observed that the number of PLA signals per cell (representing the measure for the expression of CXCR4-CCR7 heterodimers), was significantly higher in metastatic MDA-MD-231 and MDA-MD-361 cells when compared to non-invasive T47D cells in concordance with our earlier results from the FACS-FRET analysis. We next utilised the PLA approach to assess the CXCR4 and CCR7 heterodimerization in situ in human mammary epithelium from normal mammary gland tissue as well as the highly aggressive and invasive mammary metaplastic carcinoma using sections from archived paraffin-embedded tissues. The number of PLA signals representing CXCR4-CCR7 heterodimeric complexes in breast cancer tissue was dramatically increased when compared to normal breast tissue, in which CXCR4-CCR7 dimers were almost undetectable (Figure 3C). Thus, this is the first demonstration of the presence of CXCR4-CCR7 heterodimers in primary human breast cancer. Most importantly, the levels of this heterodimeric chemokine receptor, as opposed to that of its constituent protomers, is most likely responsible for the augmented CXCR4 and CCR7 functional activity in metastatic breast tumour cells, since both the CXCR4 and CCR7 individual protein expression levels were comparable between the normal and breast cancer tissues relative to the area of the mammary epithelial compartment (Supplementary Figure S3B). Together with our earlier observations in primary mammary tumour cells (Figure 2B), these results suggest that functional CXCR4 and CCR7 heterodimers are present at the later invasive stage of cancer progression. To further investigate the link between receptor heterodimerization and the clinicopathological features of breast cancer progression, we performed a PLA analysis on a breast cancer tissue microarray (TMA) containing 192 cores from archived paraffin-embedded breast tissues, encompassing all stages of breast cancer progression from normal to benign hyperplastic lesions, to ductal carcinoma in situ (DCIS), as well as invasive ductal carcinoma (IDC) including stage four tumours with distant metastasis. All tissue cores present on the TME were additionally assessed by a pathologist to confirm correct annotation and to designate tumour tissue areas for further analysis. PLA was performed using unconjugated anti-CXCR4 and anti-CCR7 antibodies, together with an Alexa Fluor® 488 anti-EpCAM antibody to distinguish epithelial tumour areas from tumour stroma for subsequent quantitation. Images of ten fields of view from each TME core, 1920 in total, were recorded and then processed by blinded PLA signal scoring using a custom designed Fiji algorithm for particle analysis. Representative photos of the H&E-stained cores from different tumour subgroups, together with composite images of the CXCR4-CCR7 dimers (PLA signals) on EpCAM-expressing mammary epithelial cells are shown in Figure 4A. Quantified numbers of PLA signals per area of breast epithelium was then correlated with the grade, stage, and lymph node status to the corresponding tumours (Figure 4B). We found that the number of CXCR4-CCR7 complexes in tissues significantly increased with tumour aggressiveness (grade, Figure 4B left panel), tumour size and spread (stage, Figure 4B middle panel), as well as the lymph node metastasis status (Figure 4B right panel). Of note, analysis of a publicly available gene expression database demonstrated that it is not the high individual expression of CXCR4 and CCR7, but their enhanced co-expression levels that significantly correlate with the increase in breast tumour grade (Figure 4C). Interestingly, the significance of gene co-expression progressively increased along the continuum of disease, suggesting potential causation. Thus, our study uncovers a clear association between cell surface expression of a CXCR4-CCR7 heterodimeric receptor through disease progression into advanced stages of mammary malignancy. This strongly suggests an important function of this unique dimeric receptor in promoting an invasive phenotype in breast cancer cells. Moreover, our data indicate that the CXCR4-CC7 heterodimeric receptor has the potential to serve as a novel biomarker for advanced stages of the breast cancer.

### 3.4. Forced Dimerization of CXCR4 and CCR7 Leads to Their Functional Activation

An important criterion in assessing GPCR heterodimerization is the demonstration of the functional significance of the complex assembly. With the view to determine if the association of the CXCR4-CCR7 complex leads to altered receptor activation in response to their cognate ligands, we utilised an FKBP-FRB based forced dimerization system (Supplementary Figure S4A), which has been extensively used to assess receptor heterodimerization on downstream signalling in various cell types, including mammary epithelial cells [40]. To this end, CXCR4- FRB (DmrC) and CCR7- FKBP (DmrA) receptor chimaeras containing synthetic ligand-binding domains were generated by subcloning the respective chemokine receptor cDNAs into expression plasmids termed CXCR4-H and CCR7-M, which enabled regulation of CXCR4-CCR7 dimer formation using AP21967 (A/C ligand heterodimerizer, ACH). Correct cell surface expression of both chimeric receptors was confirmed by FACS in transfected MDA-MB-231 cells (Supplementary Figure S4B), with similar results in T47D cells (not shown). To assess the downstream effects of CXCR4-CCR7 heterodimerization, both non-metastatic T47D and metastatic MDA-MB-231 breast cancer cell lines were co-transfected with the mixture of CXCR4-H and CCR7-M or with a control vector containing only DmrA domain, and were treated with the dimerizer drug ACH, or left untreated, followed by the addition of chemokine ligands. Forskolin induced an increase in cAMP in both cell lines and ACH treatment did not further enhance this response (Supplementary Figure S4C). 

**Figure 5:**
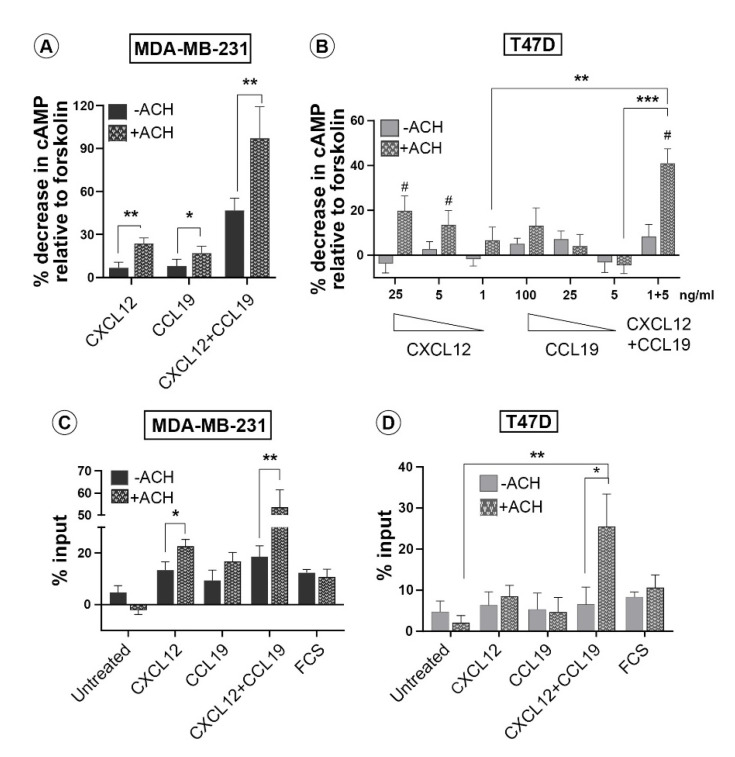
**Forced dimerization of CXCR4 and CCR7 leads to their functional activation.** Metastatic MDA-MB-231 and non-invasive T47D cell lines were co-transfected with vectors expressing heterodimerization domain–fused CXCR4 and CCR7. (**A**,**B**) cAMP relative concentration was assayed in lysates from transfected cells pre-treated with forskolin and further stimulated with CXCL12, CCL19 or their combination with or without the addition of the dimerizer drug ACH as indicated. (**C**,**D**) Matrigel transwell invasion assay was performed with transfected cells that were left untreated or treated with ACH drug as indicated. Plotted are percentages of the total cell input of 5 × 10^4^ cells per well, that have migrated through Matrigel to the underside of the transwell filter. All data shown are mean ± SEM with a two-tailed student *t*-test and are representative of three independent experiments. Levels of significance * *p* ≤ 0.05, ** *p* ≤ 0.01, *** *p* ≤ 0.001, #—significant decrease in cAMP relative to untreated cells.

The ability of CXCL12, CCL19 either alone, or in combination, to inhibit the forskolin-induced increase in cAMP was then examined in both cell lines. Forced heterodimerization of CXCR4 and CCR7 in metastatic MDA-MB-231 cells led to statistically significant decreases in forskolin-induced cAMP levels in response to CXCL12 and CCL19, either alone or in combination (Figure 5A). The effect of forced dimerization in T47D cells was more apparent in the response to CXCL12 than in CCL19 (Figure 5B). However, in combination forced dimerization uncovered a statistically significant enhancement of inhibition of forskolin-induced cAMP levels. Together, these data show that CXCR4 and CCR7 heterodimer activity can be induced in non-metastatic cells.

The capacity to invade surrounding tissues and migrate in response to stimulation is an important property of invasive tumour cells. To explore the impact of the CXCR4/CCR7 heterodimerization on the invasive capability of breast cancer cells, a Matrigel invasion assay was performed. Double-transfected ACH-treated or untreated MDA-MB-231 and T47D cells were seeded in low serum media on top of the Matrigel-coated transwells and migration towards CXCL12 or CCL19, either alone or in combination was assessed. A small increase in invasion rate was observed in transfected MDA-MB-231 cells towards CXCL12 but not CCL19 after the addition of the dimerizer drug (Figure 5C). However, upon the addition of the ACH, stimulation by both CCL19 and CXCL12 together resulted in a gain in cellular invasiveness of greater than 50% of cell total input. No chemotactic response to individual ligands was detected in double-transfected ACH-treated non-metastatic T47D cells. However, forced CXCR4-CCR7 dimerization led to the acquisition of strong invasive potential in response to CXCL12 and CCL19 in combination as a statistically significant proportion of these cells was able to migrate through Matrigel in response to combined CXCL12 and CCL19 (Figure 5D). The formation of the dimer as a prerequisite for the functional activation of CXCR4 and/or CCR7 was confirmed through control experiments with T47D cells co-transfected with CXCR4-H and control vector containing only DmrA domain. No activity of either receptor was detected in these cells in all conditions tested (Supplementary Figures S4D,E). These results show for the first time that CXCR4 and CCR7 signalling activity and invasive phenotype is specifically induced in non-metastatic cells by dimerization of these receptors.

## 4. Discussion

The fact that class C GPCRs function as heterodimers and that their context-dependent heterodimerization is critical for receptor function is widely accepted. But the significance of the oligomeric status of most numerous class A and B receptors, which constitute >90% of all GPCRs, remains hotly debated. In this manuscript, we have described the first functional CXCR4-CCR7 chemokine receptor, which is specifically expressed in advanced breast cancer cells in vitro in continuous cell lines, ex vivo, in primary mammary mouse tumour cells, human tumour cells and archived human patients breast cancer tissues. Most importantly, we have demonstrated that the function of its protomers is strictly controlled by the formation of the dimer, as neither CXCR4 nor CCR7 activation could be detected in non-invasive mammary tumour cells, where the presence of the heterodimeric CXCR4-CCR7 receptor was not found. It is important to note that even though it is widely accepted that resonance transfer approaches allow identification of interacting units within protein complexes, these techniques do not allow to sufficiently differentiate between closely located protomers versus bona fide protein aggregates. Therefore, we have employed several alternative methods to confirm the CXCR4 and CCR7 association.

Forced dimerization of the CXCR4 and CCR7 in non-invasive cells has led to a partial restoration of the functional response to their cognate ligands suggesting an involvement of other factors in addition to dimer formation in controlling activation of these chemokine receptors. Our observations thus provide the first clear evidence for a specific novel link between the CXCR4-CCR7 heterodimerization, CXCR4 and CCR7 function and the metastatic propensity of breast cancer cells.

CXCR4 protein expression has long been suggested as a survival prognostic marker in numerous cancers [41]. In contrast, CCR7 expression levels on the surface of cancer cells have not yet been sufficiently analysed to make statistically unbiased conclusions regarding correlations with cancer outcomes. However, neither of these cellular receptors has been conclusively demonstrated as a biomarker for locally invasive or metastatic tumours. Using a tailored PLA approach, we have shown for the first time that the expression of the CXCR-CCC7 dimeric complex significantly correlates with the presence of lymph node metastasis in human mammary tumours which indicates that this chemokine receptor heterodimer may be a novel biomarker of the distant spread in breast cancer. Of particular interest also is the fact that our analysis of the publicly available gene data sets showed that while individual expression levels of *CXCR4* and *CCR7* did not correlate with any breast tumour characteristics, *CXCR4* and *CCR7* co-expression was highly significantly linked to the tumour grade, further emphasizing the role of the interaction between the two receptors in breast cancer progression. 

As indicated above, forced dimerization of the CXCR4 and CCR7 demonstrated that simply bringing the receptors together under the conditions employed only partially activated their signal transduction. This finding can be interpreted in a number of ways. First, it could indicate the importance of a specific tertiary structure of the dimeric receptor for its full activity and this required specific heterodimer conformation may not be completely reproduced by just bringing two protomers together through their C-termini forced interaction. Second, our data demonstrating the complete inactivity of CXCR4 and CCR7 receptors in non-metastatic cells paints a more complex picture suggesting that inherent differences in breast cancer cells are likely to be important determinants of CXCR4-CCR7 receptor heterodimerisation in the context of tumour progression. The changes of multiple factors occurring in metastatic cells may likely be required for maximal CXCR4-CCR7 heterodimer activity. These may include the presence or absence of co-factors that change receptor conformation upon binding, expression of specific protein-modifying enzymes that mediate receptor post-translational modifications, differential expression of G-protein subunits that can selectively mediate CXCR4 and CCR7 allosteric changes or numerous other changes in cellular components that have been demonstrated to have an impact on the multimeric GPCRs [42]. We have previously found that in a panel of human breast cancer cell lines, the coupling of Gα_i_ and Gβ proteins with CXCR4 varies significantly between cell lines with different invasive properties [27]. These findings suggest that the composition of the heterotrimeric complex, together with other factors may determine the properties of chemokine receptor heteromers. 

Overall, our findings further demonstrate that chemokine receptor activity is regulated at multiple molecular levels with heterodimerization being a very significant and efficient molecular switch mechanism, which likely can be further affected by spatial and temporal protomer and accessory protein expression. This multilayered and multifactorial organisation emphasizes the potential importance of the tight control of the chemokine receptor activity in homeostasis. In pathology, a breakdown in those control mechanisms may unleash strong responses augmenting and even superseding negative regulators to advance disease progression. In cancer, inherent genetic instability selection pressure may lead to the expansion of cells with a more aggressive phenotype, which in part may be characterised as well as driven by functional CXCR4-CCR7 dimers on the cell surface. Future studies should focus on elucidating the CXCR4-CCR7 molecular dimerizer “switch” that could be then targeted for more effective therapies in metastatic breast and potentially other cancers.

## Data Availability

Data for human *CXCR4* (GC02M136114) and *CCR7* (GC17M040556) gene co-expression analysis was obtained from the Oncomine™ platform (https://www.oncomine.org/, accessed on 15 August 2021) using Curtis breast mRNA cohort.

## Supplementary Materials

The following are available online at https://www.mdpi.com/article/10.3390/life11101049/s1, Figure S1: CXCR4 and CCR7 surface expression levels are similar across breast cancer cell lines as assessed by flow cytometry, Figure S2: FACS-FRET analyses, Figure S3: CXCR4 and CCR7 protein expression levels are similar in normal mammary and primary human breast cancer tissues, Figure S4: Forced dimerization analyses.
