# Supplementary material for: CXCR4-CCR7 Heterodimerization Is a Driver of Breast Cancer Progression
Source: Life (Basel). 2021 Oct 7;11(10):1049. doi: 10.3390/life11101049 (PMC8538406; doi:10.3390/life11101049)
Supplement: Supplementary file 1 [file life-11-01049-s001.zip › life-1372542-supplementary.pdf]

life

MDPI

Supplementary Materials: CXCR4-CCR7 Heterodimerization is a Driver of Breast Cancer Progression

Valentina Poltavets , Jessica W. Faulkner , Deepak Dhatrak , Robert J. Whitfield, Shaun R. McColl and Marina Kochetkova

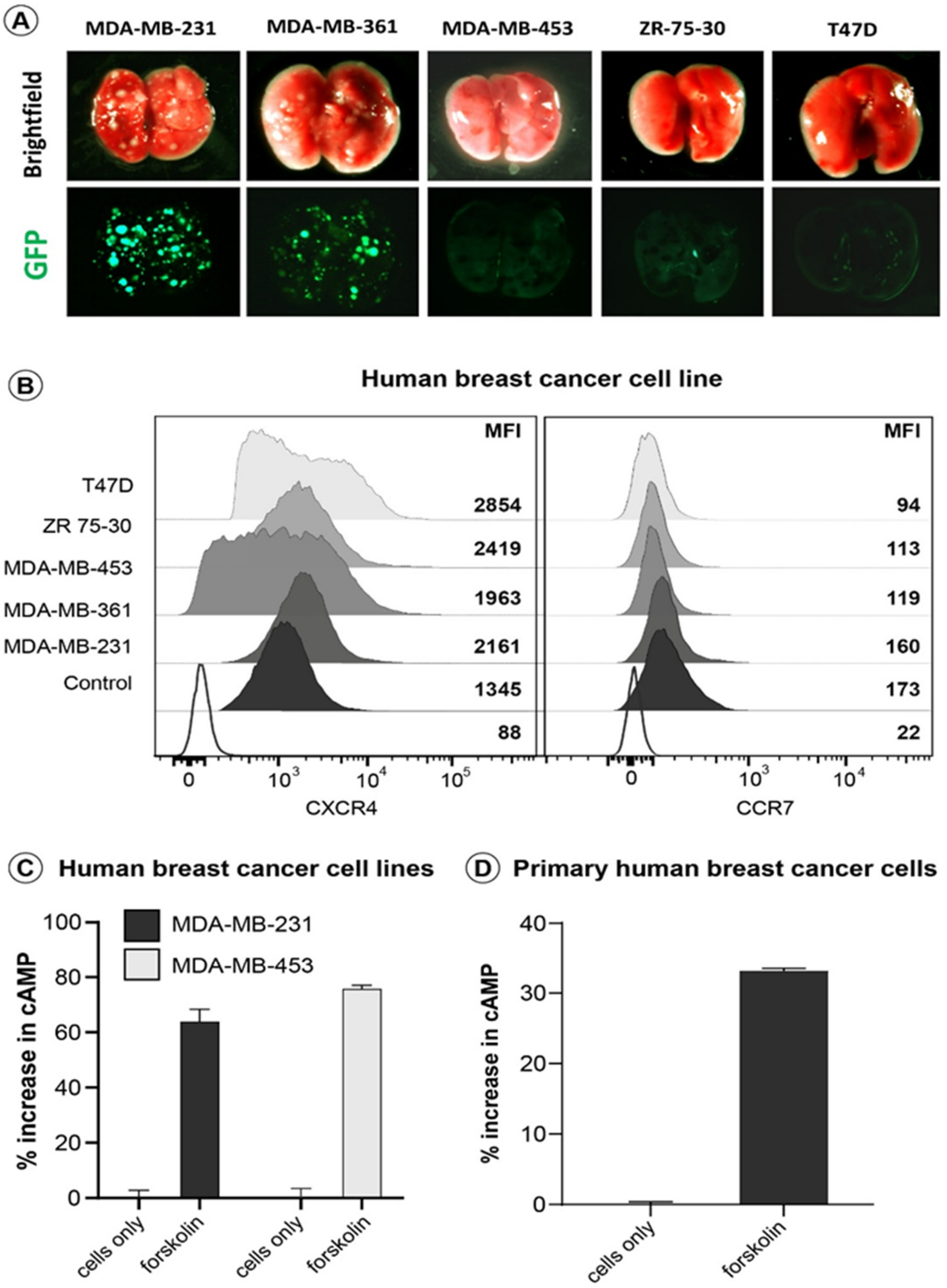

Figure S1. Invasive potential of human breast cancer cell lines is defined by their ability to form experimental lung metastasis. **(A)** Representative brightfield images (top panel) and corresponding fluorescent images (bottom panel) of lungs with metastatic lesions harvested from SCID female mice injected with GFP-labelled cell lines as indicated **(B)** CXCR4 and CCR7 surface expression levels are similar across breast cancer cell lines as assessed by flow cytometry. Mean Fluorescent Intensity (MFI) of receptor expression is indicated for each cell line. Breast cancer cell lines **(C)** and primary human tumour cells **(D)** upregulate cAMP production in response to forskolin.

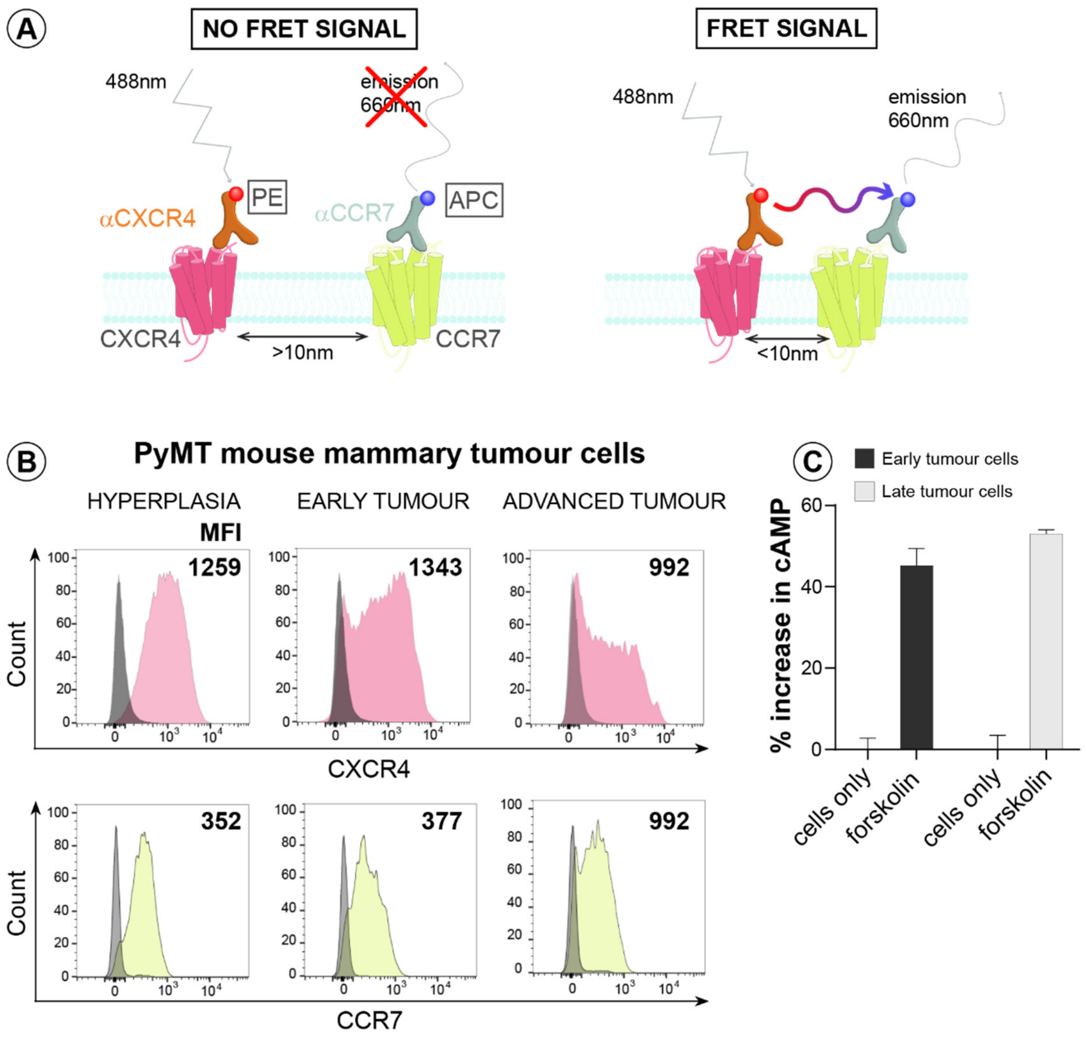

Figure S2. (A) Schematic representation of the FACS-FRET assay. **(B)** CXCR4 (top) and CCR7 (bottom) receptor expression was analysed by flow cytometry in primary tumour cells from PyMT transgenic mice at three stages of the mammary cancer progression as indicated. Shown are representative flow cytometry histograms with Mean Fluorescent Intensity (MFI) value indicated for each receptor at every tumour stage. Data are representative of at least two independent experiments. **(C)** Mouse mammary cancer cells from both early and advanced tumours upregulate cAMP production in response to forskolin.

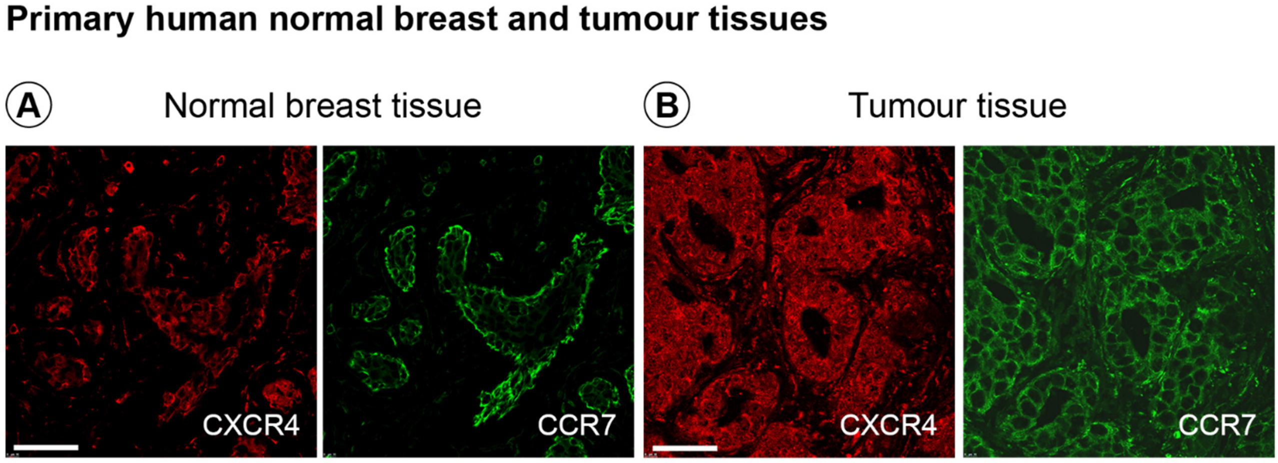

Figure S3. CXCR4 and CCR7 protein expression levels are similar in normal mammary **(A)** and primary human breast cancer **(B)** tissues. Shown are representative immunofluorescence images of tissue sections with anti-CXCR4 and anti-CCR7 antibody clones (M&M section) that were used for PLA, scale bar=50 μm.

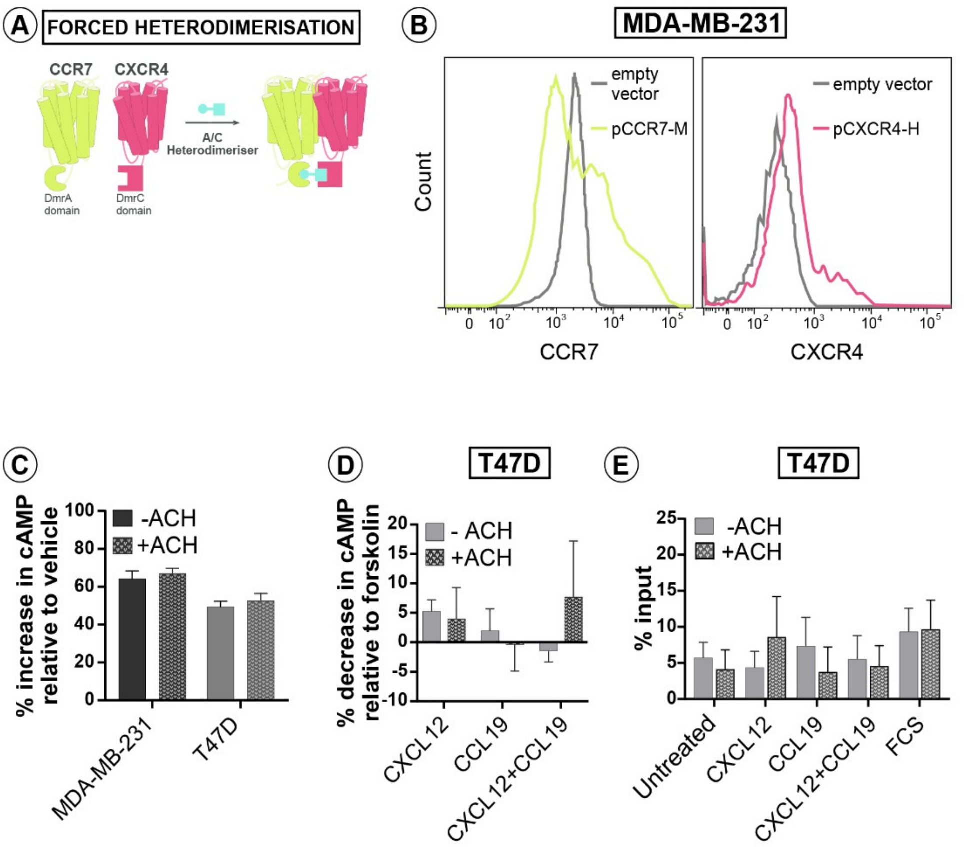

Figure S4. (A) Schematic representation of the forced dimerization approach. **(B)** Surface expression of CXCR4 and CCR7 receptors fused to dimerization domains was assessed by flow cytometry in MDA-MB-231 cells transfected with respective expression vectors relative to empty vector control. **(C)** Transfected breast cancer cells upregulate cAMP production in response to forskolin. T47D cells were co-transfected with expression vector for heterodimerization domain – fused CXCR4 and vector expressing dimerization domain only. cAMP relative concentration **(D)** and migration through Matrigel **(E)** were assessed in transfected cells after stimulation with CXCL12, CCL19 or their combination with or without addition of the dimerizer drug ACH as indicated .
